# Personalized treatment of Sézary syndrome by targeting a novel *CTLA4*:*CD28* fusion

**DOI:** 10.1002/mgg3.121

**Published:** 2014-11-27

**Authors:** Aleksandar Sekulic, Winnie S Liang, Waibhav Tembe, Tyler Izatt, Semyon Kruglyak, Jeffrey A Kiefer, Lori Cuyugan, Victoria Zismann, Christophe Legendre, Mark R Pittelkow, John J Gohmann, Fernando R De Castro, Jeffrey Trent, John Carpten, David W Craig, Timothy K McDaniel

**Affiliations:** 1 Mayo ClinicScottsdale, Arizona; 2 Translational Genomics Research InstitutePhoenix, Arizona; 3 Illumina Inc.San Diego, California; 4 Lexington Oncology AssociatesLexington, Kentucky; 5 Dermatology Associates of KentuckyLexington, Kentucky; 6 Derby Lane FarmVersailles, Kentucky

**Keywords:** *CD28*, *CTLA4*, Sézary syndrome

## Abstract

Matching molecularly targeted therapies with cancer subtype-specific gene mutations is revolutionizing oncology care. However, for rare cancers this approach is problematic due to the often poor understanding of the disease's natural history and phenotypic heterogeneity, making treatment of these cancers a particularly unmet medical need in clinical oncology. Advanced Sézary syndrome (SS), an aggressive, exceedingly rare variant of cutaneous T-cell lymphoma (CTCL) is a prototypical example of a rare cancer. Through whole genome and RNA sequencing (RNA-seq) of a SS patient's tumor we discovered a highly expressed gene fusion between *CTLA4* (cytotoxic T lymphocyte antigen 4) and *CD28* (cluster of differentiation 28), predicting a novel stimulatory molecule on the surface of tumor T cells. Treatment with the CTLA4 inhibitor ipilimumab resulted in a rapid clinical response. Our findings suggest a novel driver mechanism for SS, and cancer in general, and exemplify an emerging model of cancer treatment using exploratory genomic analysis to identify a personally targeted treatment option when conventional therapies are exhausted.

## Introduction

The emergence of molecularly targeted drugs and genomic sequencing has enabled a patient-specific cancer treatment approach whereby oncogenic somatic mutations are identified in a patient's disease and used to guide treatment (Puente et al. 2011; Tiacci et al. 2011; Stephens et al. 2012). We applied this approach to a patient with late-stage Sézary syndrome (SS), a rare, aggressive, leukemic variant of cutaneous T-cell lymphoma (CTCL) (Li et al. 2012). In SS, malignant T cells circulate in the blood and infiltrate the skin, resulting in profound redness and debilitating itch. In advanced disease, lymph node involvement and cutaneous tumor development can occur (Olsen et al. 2011). Data on the efficacy of treatments for SS are sparse, reflecting the low incidence and, until recently, the lack of standardized diagnostic, staging, and therapy evaluation schemes (Olsen et al. 2011).

## Materials and Methods

### Clinical description and tumor specimen collection

The patient was a 67-year-old Caucasian female with stage IVA SS. She initially presented 8 years earlier with a pruritic erythematous eruption on the trunk, which varied in severity over 14 months. The eruption became more consistent and diffuse and CTCL was diagnosed based on skin biopsy histology (Fig. S1). The patient subsequently developed left axillary adenopathy; flow cytometry studies of peripheral blood showed an abnormal T-cell population with reduced CD7 and CD2 expression and a CD4:CD8 ratio of ∽7:1, consistent with the diagnosis. Six years later, the patient rapidly developed innumerable, mostly ulcerated cutaneous tumors with characteristic histology. Over the disease course, multiple treatments were attempted and ultimately failed, including narrow-band ultraviolet B radiation, extracorporeal photopheresis, interferon alpha 2a, bexarotene, suberoylanilide hydroxamic acid, interferon gamma, interferon alpha 2b, gemcitabine, local radiotherapy, romidepsin, and PEGylated liposomal doxorubicin (Olsen et al.).

At the time of tumor tissue collection, the patient had generalized erythroderma, malaise, and intractable pruritus as well as innumerable firm, dome-shaped skin tumors, some of which were ulcerated. Two nonulcerated tumors, one from the right thigh and the other from the lower back, were collected as 8-mm punch biopsies. Histologic examination of biopsied tumors confirmed the presence of a diffuse superficial and deep dermal infiltrate of atypical T lymphocytes, consistent with the diagnosis of tumor-stage SS. Saliva, as a source of normal constitutional DNA, was collected from the patient using an Origene DNA saliva collection kit (DNA Genotek, Inc., Kanata, Ontario, Canada).

### Informed consent

Prior to DNA sequencing in a CLIA-certified, CAP-accredited laboratory the patient signed a consent form outlining psychological, privacy, and other risks of genomic sequencing. For RNA sequencing (RNA-seq) analysis, the patient provided written informed consent into TGen's IRB approved Protocol for Biospecimen Banking and Cancer Research (WIRB protocol #20110843).

### Laboratory methods

DNA and RNA isolated from the tumors were subjected to whole genome DNA and RNA sequencing using standard library preparation methods, instrumentation, and analysis software pipelines as described below. As controls, DNA from the patient's saliva and RNA from CD4+ T cells from two unrelated donors were analyzed in parallel.

### Nucleic acid preparation

DNA and RNA from tumors were isolated using Qiagen's AllPrep kit (Qiagen, Germantown, MD, USA). Germline DNA was isolated using Origene's DNA saliva collection kit.

### Library preparation and sequencing

WGS (whole genome sequencing) (Bentley et al. 2008) of genomic DNA from the two tumors and saliva was performed by the Illumina Clinical Services Laboratory using a Paired-End Library Preparation kit. Libraries were clustered using V2 Paired-End Cluster Generation Kits on a Cluster Station and sequenced on a GA IIx (2 × 121 bp) with a Paired-End Module and an SBS Sequencing Kit v5 (Illumina, San Diego, CA, USA). Ten nanograms of total RNA for each sample (two tumors and two controls) were used to generate separate whole transcriptome libraries using the Nugen Ovation RNA-Seq System v2 and Illumina's TruSeq DNA Sample Preparation Kit. An equimolar pool of all four barcoded libraries was clustered on the cBot using the TruSeq PE Cluster Kit v3 and sequenced on the HiSeq 2000 (101 × 7 × 101; Illumina).

### Data analysis

Raw DNA and RNA sequence was processed into reads using CASAVA software (Illumina). DNA was aligned to the human reference genome (build 36) using Illumina's ELAND2 pipeline, followed by somatic mutation analysis to identify single-nucleotide variants (SNVs), indels, and copy number variants (CNVs; Fig. S2) as described previously (Craig et al. 2013). For RNA-seq analysis, reads were aligned to the human genome using Tophat 1.2.0 (Trapnell et al. 2009) followed by transcript assembly/abundance estimate, and differential expression analysis with Cufflinks 0.9.3 (Trapnell et al. 2010). Sequencing data can be accessed through NCBI's (National Center for Biotechnology Information) dbGaP (database of Genotypes and Phenotypes) under accession number phs000773.v1.p1.

## Results and Discussion

### Sequencing analysis

Sequencing metrics are shown in Table S1. CNV analysis of genomic data indicated widespread chromosomal instability (Fig. S2). To prioritize potentially functionally relevant somatic events, we focused on aberrations present in both tumors for which there was strong physical evidence of functional alteration as inferred from: (1) loss of both copies (*N* = 6 genes in two regions of loss); (2) in-frame gene fusion (*N* = 2 genes in one event); (3) intragenic deletion (*N* = 1 gene); (4) deletion of one allele coupled with a coding mutation in the second (*N* = 1 gene). Although screening was performed irrespective of function, seven of the nine genes (Table1) have functions consistent with a potential role in cancer. Four are oncogenes or tumor suppressor genes (*CDKN2A*, *CDKN2B* [Laharanne et al. 2010], *PARK2* [Morris et al. 2010], and *WAPAL* [Kueng et al. 2006]), and three, described below, regulate T-cell proliferation.

**Table 1:** Significant^1^ somatic copy number variation mutations identified through WGS

Region	Type	Genes	Gene functions
Chr2: 204,596,010-204,736,442	Gene fusion/amplification	*CTLA4, CD28*	Transmembrane repressor and activator of T-cell activation (respectively); Reviewed in Laharanne et al. (2010)
Chr 10: 88259540	Point mutation in highly conserved region coupled with deletion of other allele.	*WAPAL*	Regulates cohesin ring dissociation during sptation (Kueng et al. 2006); mutations are associated with chromosomal instability (Ohbayashi et al. 2007)
Chr 5: 141470000	Homozygous deletion	*GNPDA*^2^ *NDFIP1*	NDFIP1 is necessary for the catalytic activity of the ubiquitin protein ligase Itch (Mund and Pelham 2009), which mediates CTLA4-induced inhibition of T-cell activation (Hoff et al. 2010)
Chr 9: 21863000- 21909000	Homozygous deletion	*MTAP*^2^*, CDKN2A, CDKN2B*	CDKN2A and CDKN2B are tumor suppressor genes previously reported as deleted in SS (Laharanne et al. 2010)
Chr6:762,667,533-762,752,701	Heterozygous intragenic deletion (removes exon 3)	*PARK2*	Tumor suppressor gene in glioblastoma (Veeriah et al. 2010) and colorectal cancer (Poulogiannis et al. 2010)

WGS, whole genome sequencing.

1 ”Significant” mutations are those for which there was strong physical evidence of functional alteration or inactivation in both tumors. Note that the T-cell receptor alpha and gamma genes were also identified by these criteria on the basis of homozygous chromosomal deletion; these genes are not included in this table as the deletions are expected to have occurred as part of normal T-cell development, and not as part of carcinogenesis.

2 *GNPDA* and *MTAP* have no obvious connection to cancer or T-cell biology and are presumably passengers of the deletions that removed *NDFIP* and *CDKN2A/CDKN2B*, respectively.

**Figure 1:**
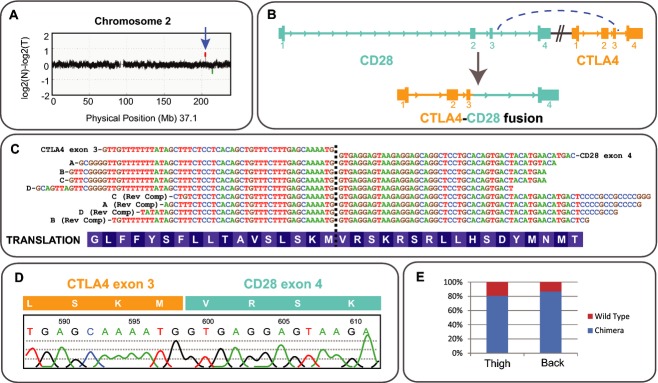
Identification of an amplified *CTLA4-CD28* fusion (A) CNV plot of chromosome 2. The identified amplification (blue arrow) contains only the two fused genes, *CTLA4* and *CD28*. The *y*-axis indicates the normalized log2 fold difference in copy number between the normal and tumor samples, inferred from sequencing read depth. (B) *CD28* and *CTLA4* loci and the chimeric product of gene fusion. (C) RNA-seq reads confirmed an in-frame fusion between *CTLA4* and *CD28*. The breakpoint is marked with a dotted line. (D) Sanger sequencing validation of a reverse transcribed copy of the fusion transcript. The chromatogram shows the junction between *CTLA4* and *CD28*. (E) The chimeric *CTLA4-CD28* transcript is the predominant form of *CTLA4*RNA expressed in the examined tissues. This is despite the fact that ∽40% of the tumor is normal infiltrate, which is expected to express wild-type *CTLA4*.

Given that SS is a cancer of T cells, a striking mutation was an amplified fusion on chromosome 2, comprising two adjacent genes encoding the key opposing regulators of T-cell proliferation, cytotoxic T lymphocyte antigen (*CTLA4*) and *CD28* (Fig.1A). The fusion (Fig.1B) predicts a novel in-frame chimeric transcript encoding the extracellular and transmembrane domains of CTLA4, joined to the intracellular signaling domain of CD28. RNA-seq revealed abundant transcription of a spliced, in-frame fusion (Fig.1C), which was validated by Sanger sequencing (Fig.1D). The fusion was the predominantly expressed form (Fig.1E).

**Figure 2:**
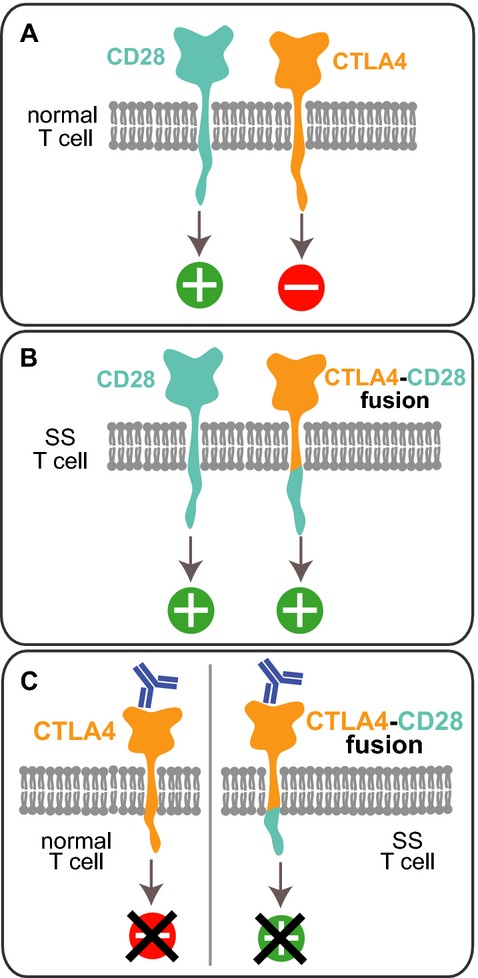
DNA and RNA sequencing evidence of CTLA4-CD28 fusion (A) In normal T cells, activation of CD28 stimulates proliferation, whereas activation of CTLA4 inhibits. (B) In SS T cells expressing the chimera, CTLA4 activation would aberrantly stimulate proliferation through the intracellular CD28 domain. (C) Model of ipilimumab's inhibition of SS proliferation. In normal cells (left), binding of ipilimumab to CTLA4 blocks the inhibitory CTLA4 signaling. In SS cells (right), ipilimumab is predicted to inhibit proliferation by blocking the aberrant stimulatory signaling delivered by the chimeric protein.

In normal T cells, CD28 provides the key T-cell costimulatory signal during activation. Its engagement leads to a cascade of events, including cellular proliferation and *CTLA4* transcription. Once expressed, CTLA4 inhibits proliferation by opposing the effects of CD28 (Fig.2A) (Krummel and Allison 1995). In the chimera, the inhibitory cytoplasmic tail of CTLA4 was replaced by the activating tail of CD28. This chimera is predicted to provide an aberrant stimulatory signal (Fig.2B) suggesting a novel mechanism contributing to oncogenic proliferation. In addition to this fusion, a second hit to the CTLA4 pathway occurred through a homozygous deletion of the key CTLA4 signal mediator, *NDFIP1* (Fig. S3), which was under-expressed in both tumors (ln(fold) = −1.10, mean *P* = 1.58E-04). The product of this gene is necessary for catalytic activity of the ubiquitin ligase Itch (Mund and Pelham 2009). In normal T cells, Itch mediates CTLA4-induced inhibition of T-cell activation (Hoff et al. 2010) by targeting for degradation growth signaling molecules activated by stimulation of T-cell receptor and CD28 (Shembade et al. 2008; Mund and Pelham 2010; Ahmed et al. 2011). Thus, through inactivation of Itch, the deletion of *NDFIP1* is predicted to act as a functional knockout of the remaining *CTLA4* allele.

**Figure 3:**
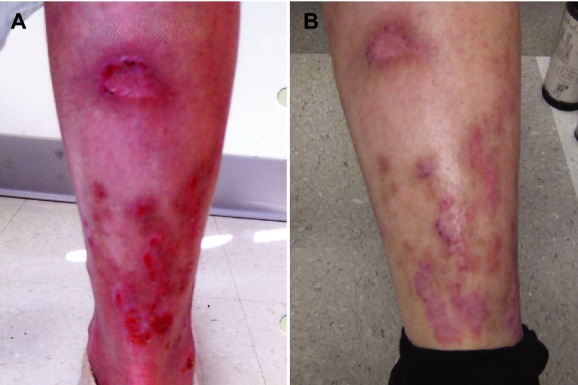
Clinical response to ipilimumab (A) A pre-ipilimumab photo of the patient's calf is shown and displays generalized erythroderma and ulcerated cutaneous tumors. (B) Following ipilimumab treatment, the patient experienced a reduction in pruritus and erythema as well as healing of ulcerated tumors and a decrease in overall tumor number and size.

With progressing disease and no further rational therapeutic candidates, the patient was treated by blockade of the chimeric CTLA4-CD28 protein using the anti-CTLA4 monoclonal antibody ipilimumab, an FDA-approved antimelanoma drug (Fig.2C). The patient received four doses (3 mg/kg, every 3 weeks) and experienced no obvious toxicities. Within 10 days of administration, she demonstrated a marked clinical response including 50% reduction in erythema, 75% size reduction of dermal and subcutaneous tumors with 50% size reduction of lower leg ulcers (Fig.3), and self-reported decrease in itching. The patient's energy level markedly increased, enabling resumption of normal life activities. By the sixth week of therapy, despite continued improvement in erythema and energy levels, she rapidly developed skin tumors on the head and neck, histologically consistent with CTCL. The disease progressed rapidly to death 3 months after the last dose.

## Conclusions

The finding of a *CTLA4-CD28* fusion in the cancer cells from an SS patient is, to our knowledge, novel. Furthermore, the general mechanism of cancerous growth being driven by a negative regulator of proliferation that has been converted into a positive regulator through fusion of positive signaling domain is, to our knowledge, novel to the study of cancer as a whole. While it will be important to confirm this hypothesized mechanism through cellular and biochemical studies of the identified fusion, one could argue that this has already been done in principle through experiments previously conducted, without reference to SS, to elucidate the general mechanisms regulating T-cell proliferation. Several laboratories have generated synthetic chimeras containing CTLA4's extracellular domain fused to CD28's cytoplasmic domains (Yin et al. 2003; Dennehy et al. 2006). Of importance to this report, expressing these fusions in cultured cells triggers antigen-independent CD28 signaling in response to CTLA4 engagement, demonstrating the reversed signaling polarity we propose to be active in this case of SS. Although the *CTLA4-CD28* fusion in the patient's tumors involved only one *CTLA4* allele, and we see no evidence of damage to the remaining *CTLA4* allele, we suggest, based on the patient's response, that the homozygous *NDFIP1* deletion acts as a functional hit to the remaining *CTLA4* allele, ensuring that the ectopic CD28 signaling from the chimeric *CTLA4* allele acts unopposed. Supporting this notion, knockout mice with targeted deletions of *NDFIP1* (Oliver et al. 2006) or its downstream mediators *CTLA4* (Khattri et al. 1999) and *ITCH* (Perry et al. 1998) suffer from T-cell-mediated disorders, many resembling SS symptoms, including chronic skin inflammation and pruritus.

The tragic and rapid disease progression following the initial period of response observed in this patient is a recurring theme in many molecularly targeted therapies. Treatment may select for growth in permissive niches, or for cell subpopulations containing compensating mutations. In this case, it is also possible that the drug's labeled dosage and timing (approved for melanoma, not SS) were suboptimal for this indication. These possibilities underscore the need for additional laboratory studies, including surveying other SS patients for *CTLA4-CD28* fusions, and, if found, consideration of emerging strategies for clinical trials in this rare cancer (Tan et al. 2003).

Our findings highlight the clinical utility of unbiased mutational analysis of tumors using genomic sequencing. The paradigm in rare cancers, where large-scale clinical trials are challenging, is moving toward identifying personal somatic alterations to guide genomically enabled treatment decisions.

## Supporting Information

**Table S1.** Whole genome and RNA sequencing metrics. **Figure S1.** Patient tumor histology. (A) A neoplasm composed predominantly of T cells extending throughout the dermis into subcutaneous fat is shown (20×). (B) Atypical T lymphocyte-forming Pautrier's abscesses in the overlying epidermis is shown (400X). **Figure S2.** Copy number variation analysis. A CNV plot is shown (next page). Each panel represents an individual chromosome. The *y*-axis indicates the log2 fold difference in copy number, as inferred from sequencing read depth, between the normal and tumor samples. Overall, CNV analysis revealed regions of both chromosomal gains (red shading, upward signal deflection, encompassing 1437 genes) and losses (green, downward signal deflection, 1115 genes) across the sequenced tumor genomes. Detected aberrations range from single copy gains or losses events spanning entire chromosomes (e.g., chromosomes 3 and 7), chromosome arms (e.g., long and short arms of chromosome 8 and short arms of 17), as well as other events spanning many megabases (chromosomes 6, 9, 10, 15, 17, and 19), and those more focal events (e.g., focal amplification on chromosome 2q and deletion on chromosome 5q). Given the large number of events, we focused our analysis on genes for which there was a strong evidence of physical inactivation (Table1). **Figure S3.***NDFIP1* homozygous deletion. A close-up of the CNV plot for chromosome 5 is shown. The arrow demarcates the location of the copy number loss that was identified in whole genome data and that encompasses the *NDFIP1* gene. The *y*-axis indicates the log2 fold difference in copy number, as inferred from sequencing read depth, between the normal and tumor samples.
