# Supplementary material for: Personalized treatment of Sézary syndrome by targeting a novel CTLA4:CD28 fusion
Source: Mol Genet Genomic Med. 2014 Nov 27;3(2):130–6. doi: 10.1002/mgg3.121 (PMC4367085; doi:10.1002/mgg3.121)

**Supplementary Table**

**Table S1. Whole genome and RNA sequencing metrics**

|  | **Germline**  **(saliva)** | **Tumor 1 (thigh)** | **Tumor 2 (back)** | **T cell control 1** | **T cell control 2** |
| --- | --- | --- | --- | --- | --- |
| Total number of WGS reads | 1,458,069,355 | 1,066,743,334 | 972,353,979 |  |  |
| Total number of mapped WGS reads | 1,426,893,857 | 1,046,687,522 | 951,629,846 |  |  |
| %mapped WGS reads | 97.86 | 98.12 | 97.87 |  |  |
| Average mapped sequence coverage | 45.92 | 33.68 | 30.62 |  |  |
| Total number of RNA reads |  | 59,584,414 | 67,301,114 | 84,923,010 | 52,495,867 |
| Total number of mapped RNA reads |  | 59,584,414 | 67,301,114 | 84,923,010 | 52,495,867 |

**Supplementary Figures**

**Figure S1. Patient tumor histology** (A) A neoplasm composed predominantly of T-cells extending throughout the dermis into subcutaneous fat is shown (20X). (B) Atypical T lymphocyte forming Pautrier’s abscesses in the overlying epidermis is shown (400X).

**
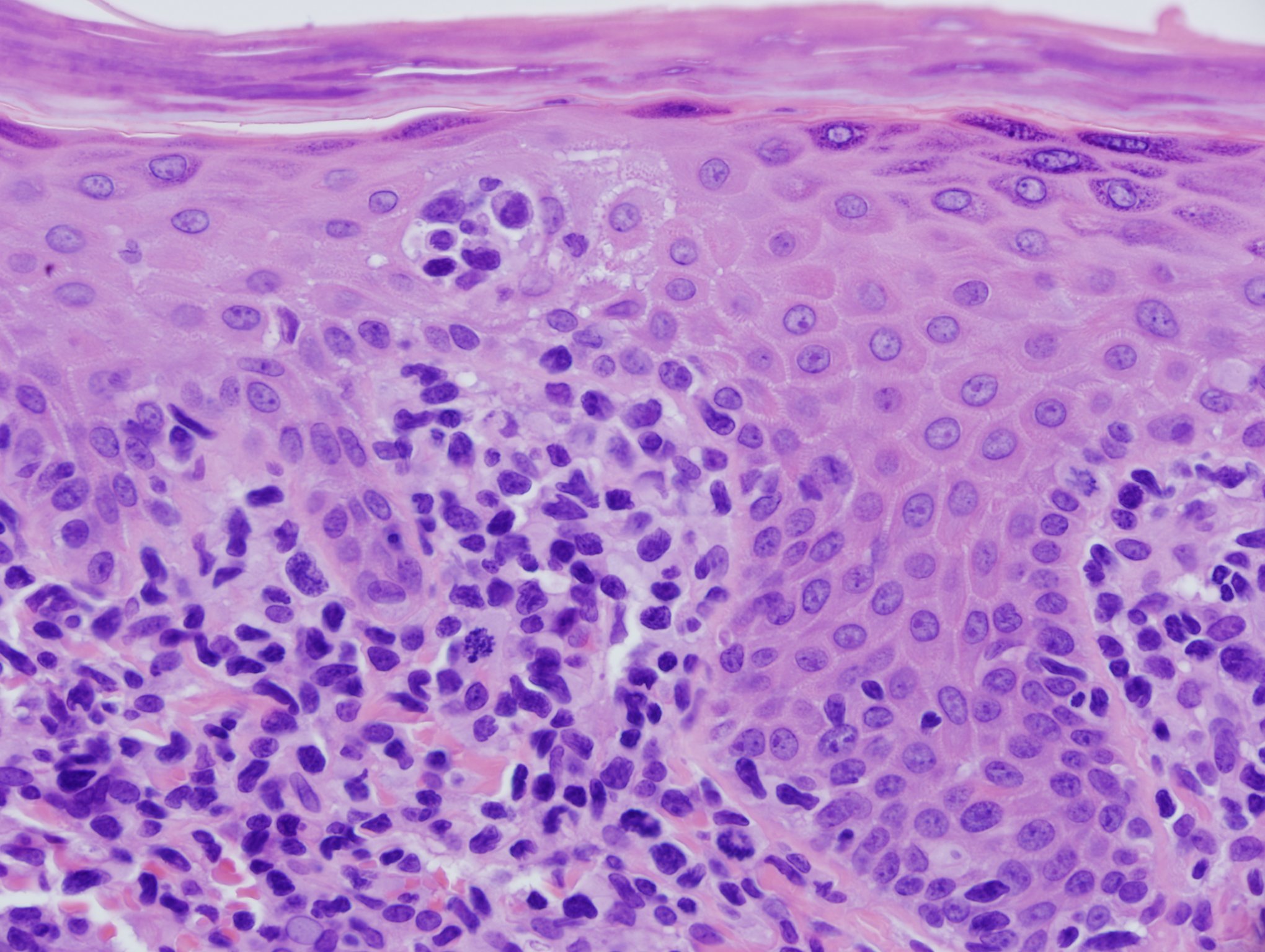

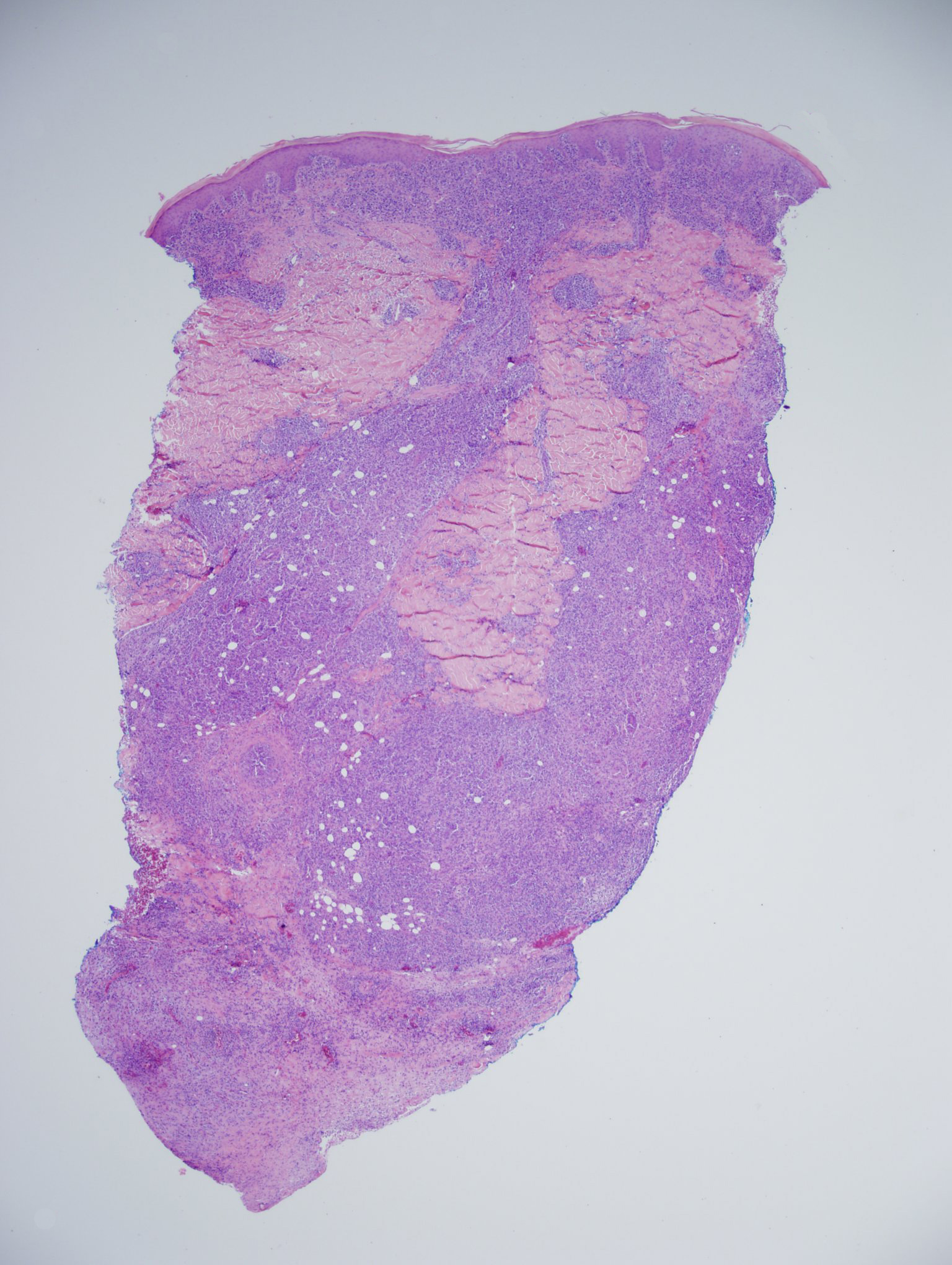
**

**B**

**A**

**Figure S2.** **Copy number variation analysis**

A CNV (copy number variant) plot is shown (next page). Each panel represents an individual chromosome. The y-axis indicates the log2 fold difference in copy number, as inferred from sequencing read depth, between the normal and tumor samples. Overall, CNV analysis revealed regions of both chromosomal gains (red shading, upward signal deflection, encompassing 1437 genes) and losses (green, downward signal deflection, 1115 genes) across the sequenced tumor genomes. Detected aberrations range from single copy gains or losses events spanning entire chromosomes (e.g., chromosomes 3 and 7), chromosome arms (e.g., long and short arms of chromosome 8 and short arms of 17), as well as other events spanning many megabases (chromosomes 6, 9, 10, 15, 17, and 19), and those more focal events (e.g., focal amplification on chromosome 2q and deletion on chromosome 5q). Given the large number of events, we focused our analysis on genes for which there was a strong evidence of physical inactivation (Table 1).


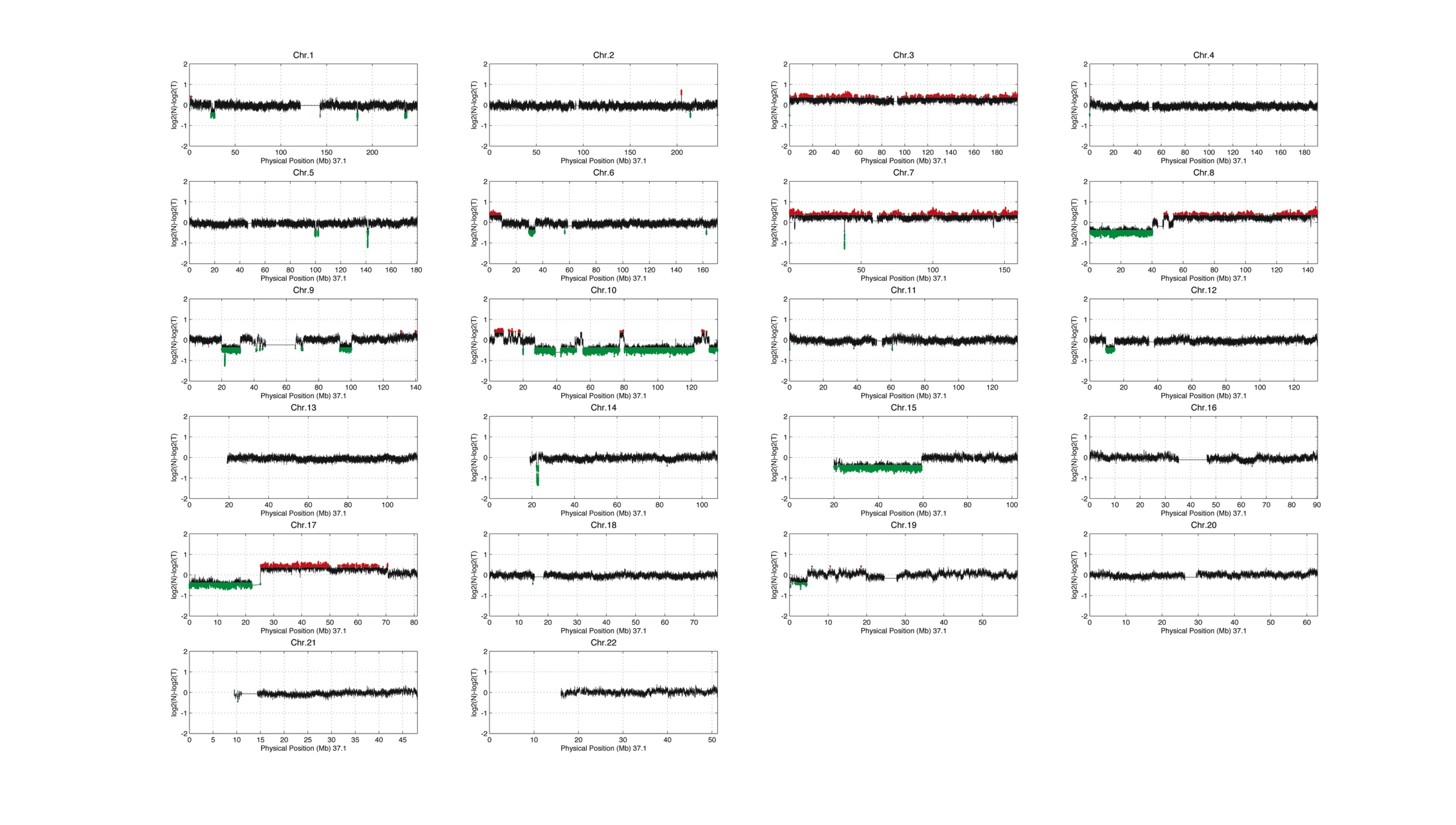


**Figure S3.** ***NDFIP1* homozygous deletion**

A close-up of the CNV plot for chromosome 5 is shown. The arrow demarcates the location of the copy number loss that was identified in whole genome data and that encompasses the *NDFIP1* gene. The y-axis indicates the log2 fold difference in copy number, as inferred from sequencing read depth, between the normal and tumor samples.


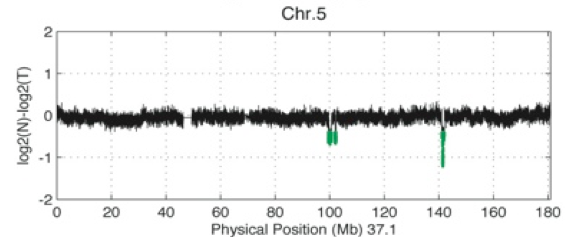

Supplement: Supplementary file 1 [file mgg30003-0130-sd1.doc]
